# Strategies for Monitoring Outcomes in HIV-Exposed Uninfected Children in the United Kingdom

**DOI:** 10.3389/fimmu.2016.00185

**Published:** 2016-05-17

**Authors:** Claire Thorne, Pat Tookey

**Affiliations:** ^1^Population, Policy and Practice Programme, UCL Institute of Child Health, University College London, London, UK

**Keywords:** HIV-exposed, fetal exposure, antiretroviral drugs, surveillance, safety

## Abstract

Surveillance of pregnancies in women living with HIV is carried out on a national basis in the United Kingdom (UK) through the National Study of HIV in Pregnancy and Childhood. There are currently around 1100–1200 HIV-exposed uninfected (HEU) infants born every year in the UK, where vertical transmission of HIV now occurs in fewer than 5 in every 1000 pregnancies. By the end of 2014, there was a cumulative total of more than 15,000 HEU children with any combination antiretroviral therapy (cART) exposure and more than 5000 with cART exposure from conception in the UK. HEU infants are increasingly being exposed to newer antiretroviral drugs for which less is known regarding both short- and long-term safety. In this commentary, we describe the approaches that have been taken to explore health outcomes in HEU children born in the UK. This includes the Children exposed to AntiRetroviral Therapy (CHART) Study, which was a consented follow-up study carried out in 2002–2005 of HEU children born in 1996–2004. The CHART Study showed that 4% of HEU children enrolled had a major health or development problem in early childhood; this was within expected UK norms, but the study was limited by small numbers and short-term follow-up. However, the problems with recruitment and retention that were encountered within the CHART Study demonstrated that comprehensive, clinic-based follow-up was not a feasible approach for long-term assessment of HEU children in the UK. We describe an alternative approach developed to monitor some aspects of their long-term health, involving the “flagging” of HEU infants for death and cancer registration with the UK Office for National Statistics. Some of the ethical concerns regarding investigation of long-term outcomes of *in utero* and perinatal exposure to antiretrovirals, including those relating to consent and confidentiality, are also discussed.

## HIV Epidemiology in the UK

There were around 104,000 people living with HIV in the UK in 2014, with an overall prevalence of 1.9 per 1000 people aged 15 years or older, based on multi-parameter evidence synthesis modeling conducted by Public Health England (1). Approximately five in six people living with HIV are aware of their diagnosis, and around 82% have been linked to HIV care. With respect to women, there were an estimated 34,400 women living with HIV in the UK in 2014, with a prevalence of 1.7 per 1000 among women aged 15–44 years. Among people with diagnosed HIV, 91% were receiving antiretroviral therapy (ART) overall, although this figure was 74% in young adults aged 16–24 (1).

## Surveillance of HIV in Pregnant Women and Their Children

Surveillance of pregnancies in women living with HIV is carried out in the United Kingdom (UK) and Ireland through the National Study of HIV in Pregnancy and Childhood (NSHPC) (www.ucl.ac.uk/nshpc). The NSHPC has continuously monitored HIV infection in pregnant women and children in the UK and Ireland since the late 1980s, and uses two population-based, active surveillance reporting schemes, one obstetric and the other pediatric. These allow comprehensive coverage of all pregnancies to HIV-positive pregnant women living in the UK or Ireland, all infants born to HIV-positive women, and all children living with HIV infection (2).

Within the obstetric scheme, a named respondent in every maternity unit in the UK and Ireland is responsible for notifying all pregnancies in HIV-positive women, regardless of timing of diagnosis. For each reported pregnancy, demographic and clinical information is obtained at notification, with outcomes subsequently collected using standardized forms. The pediatric reporting scheme involves monthly notification of HIV-exposed and infected children through the British Paediatric Surveillance Unit of the Royal College of Paediatrics and Child Health; pediatric units seeing large numbers of HIV-exposed children report directly to the NSHPC. Following the initial report, a follow-up form is sent to obtain the HIV status of exposed children. These active schemes have close monitoring and follow-up of non-response to ensure high reporting rates. Infants born in the UK or Ireland to women living with HIV should be independently reported *via* both schemes; based on data for 2000–2010, over 90% of infants were reported through both schemes.

No names are requested or recorded. Respondents provide data on demographic characteristics, laboratory results, maternal concurrent infections, ART use, obstetric management, and perinatal outcomes. Obstetric and pediatric reports are linked by date of birth, geographic location of report, National Health Service (NHS) number (a unique identifier), and other demographic variables. Consent is not required for case notification to the NSHPC, and surveillance is exclusively *via* health-care providers. To date, over 19,000 pregnancies to women living with HIV in the UK have been reported, with a further nearly 2000 from Ireland. This paper concentrates mainly on the UK data and situation.

## Exposure of Infants to Antiretrovirals in Pregnancy and/or Early Life

Routine antenatal HIV testing was introduced throughout the UK and Ireland from 1999 onward and screening uptake in England has exceeded 97% since 2011 (3). In 2014, more than 690,000 pregnant women in England were tested, with 1.5 women per 1000 testing HIV-positive (1). Mother-to-child transmission rates have been lower than 1% for several years, and in 2010–2011, the rate was 0.46% (95% confidence interval: 0.21–0.86%) (2). There are currently around 1100–1200 HIV-exposed uninfected (HEU) infants born every year in the UK, with a further 80–100 born in Ireland. By the end of 2014, there was a cumulative total of more than 15,000 HEU children with any combination antiretroviral therapy (cART) exposure and more than 5000 with cART exposure from conception in the UK.

In a recent analysis of nearly 6000 pregnancies delivered in 2009–2014, for 51%, the mother was on ART at conception, in 28%, she was diagnosed but not on ART, and in 21%, she was diagnosed with HIV for the first time during pregnancy (4). Overall, 98% of pregnancies were exposed to cART, with a further 1% exposed to monotherapy. Of the pregnancies in which cART was initiated during this time period, a significant trend toward earlier start was apparent, with the median gestation at initiation shifting from 22 weeks gestation in 2009–2011 to 20 weeks in 2012–2014.

HIV-exposed uninfected infants are increasingly being exposed to newer antiretroviral drugs for which less is known regarding both short- and long-term safety (5–8). Although the benefits of cART for maternal health and prevention of MTCT are profound, it is recognized that there is a need for continued surveillance of the safety of the use of in pregnancy, particularly any potential late effects (7, 8). Health differences in HEU children compared to unexposed children are increasingly being reported, including metabolic, mitochondrial, growth, endocrinological, immune, and hematological differences. The potential reasons behind these are likely to be complex and inter-related, and may include effects of *in utero* exposures (maternal HIV, ART, other medication, or illicit drugs), an altered microbiome, growing up in an HIV-affected family, or other factors (5–12).

## Approaches to Long-Term Follow-up of HEU Children in the UK

### The Children Exposed to Antiretroviral Therapy Study

The Children exposed to AntiRetroviral Therapy (CHART) Study, conducted between 2002 and 2005, was designed to investigate the feasibility of establishing a national, clinic-based follow-up of HEU children with ART exposure (13). This study was designed to sit alongside the core surveillance schemes of the NSHPC, which provided information on HEU children eligible for enrollment (i.e., those born between 1996 and 2000). Recruitment was initially restricted to seven hospitals in London and four hospitals elsewhere in England with known large populations of HEU children; between 2003 and the 2005 (study’s close), recruitment was expanded to all hospitals with deliveries of HEU infants. Pediatric respondents within the NSHPC were requested to approach the families of eligible children to seek consent for participation, or to contact an alternative health-care professional to do so if this was considered more appropriate. Data collection consisted of a questionnaire to be completed by the health-care professional annually, in consultation with the parent/carer of the child; this included items on height, weight, hospital admissions, specialist referrals, ongoing medication, developmental problems (speech, hearing, sight, behavior, and mobility), and any serious health problems. This information was then linked to the records held within the NSHPC on ART exposure, demographics, perinatal details, and laboratory investigations.

**Figure 1:**
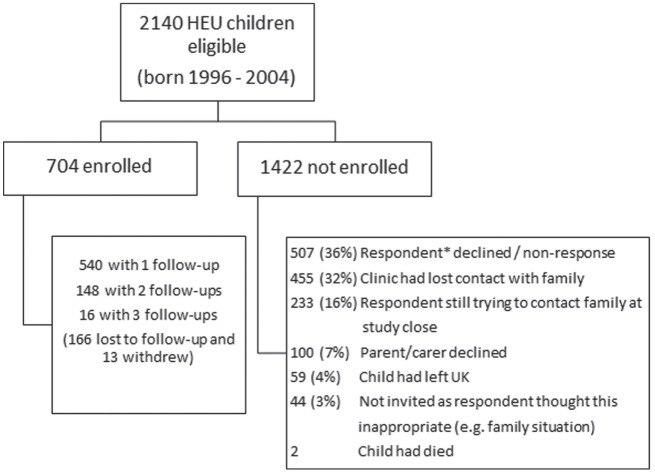
**Flowchart providing enrollment in CHART Study and reasons for non-enrollment**. *Paediatric respondent in the NSHPC.

Of around 2100 HEU children eligible for enrollment, only 34% had been enrolled by the end of the study (Figure 1). The most common reasons for non-enrollment were formal decline of the pediatric respondent to participate, with the main reason given being lack of clinic resources, or non-response of respondents who had not formally indicated non-participation. Of note, among the children whose parents or carers were approached to invite to participate, the decline rate was 12% (100/804). Comparison of the enrolled with the unenrolled groups of children showed that there were no significant differences with respect to maternal region of birth, maternal HIV risk factor, *in utero* ART exposure, or preterm delivery, although enrolled children were born to slightly older mothers.

Of the study population of 704 children, a quarter were lost to follow-up following enrollment and 2% withdrew from the study. This, combined with the slow accrual of HEU children to the study, meant that by the end of the study there was only repeated follow-up data on a quarter of children, with most just having data available at a single time point. Nearly all of the enrolled children had been exposed to ART *in utero*, with 70% exposed to cART. The median age at CHART assessment was 24 months. Overall, 63% (440/704) of the children had at least one health or developmental problem reported, the majority of which were common childhood infections or conditions. The main groups of conditions were colds/chest infections (in 201 children); wheezing/asthma, eczema, or other allergies (93 children); speech delay or problems (50 children); gastroenteritis (28 children); and febrile seizures (11 children). A further 63 children had other minor developmental or behavioral problems. There were significant health problems or conditions reported in 27 (4%) children of whom 93% were ART-exposed; 5 had with sickle cell anemia, 3 had cerebral palsy (all preterm), 3 had autistic spectrum disorder, 1 had traumatic head injury, and the remainder had various congenital defects. For the enrolled children as a whole, 9% had been admitted to hospital at least once.

Although comparison of maternal characteristics of the enrolled with the non-enrolled children was reassuring, with no significant difference with respect to specific maternal/exposure characteristics, the low enrollment rate precluded any firm conclusions on the safety of ART exposure in HEU children. Furthermore, follow-up time was short, and only a quarter of the children had repeat questionnaires available. Most of the significant health problems identified were congenital abnormalities, on which data are already routinely collected in the NSHPC. The prevalence rates of common conditions in the enrolled children were compared with those reported for the UK Millenium Cohort Study (MCS), which oversampled children from disadvantaged and minority ethnic wards (14), and the observed rates were in the range reported for the MCS.

The CHART Study demonstrated the challenges in conducting a follow-up study of HEU children, with key barriers being lack of capacity at pediatric clinics and family mobility. It was concluded that consented, clinic-based follow-up would not be a feasible approach for monitoring outcomes in ART-exposed HEU children in the UK.

### Survey of Parents’ and Health-Care Providers’ Views on Long-Term Follow-up of HEU Children

Following the experiences within the CHART Study, there was a need to explore alternative approaches to monitoring the health of HEU children and, in particular, the acceptability of different approaches to parents/carers and to health-care professionals. Two surveys were conducted with these two stakeholder groups during 2004 and 2005 to assess their views on four different strategies for follow-up (Table 1) (15). The parent/carer survey was carried out in 12 pediatric clinics and 2 genitourinary medicine clinics (8 in London, 1 in Wales, 1 in Scotland, and 4 in England), with 140 questionnaires completed, mostly by mothers of HEU children (91%). Nearly all parents/carers agreed with the statement “it is important to follow-up uninfected children to see if there are any side effects from anti-HIV drugs.” All, but one, parent/carer respondents indicated that at least one of the four follow-up strategies would be acceptable, and two-fifths supported more than one option. The most popular strategy was clinic contact (61%). Overall, 23% of respondents strongly objected to at least one of the options, with postal contact as the option that was most strongly objected to. Eight percent of parents/carers strongly objected to the “no direct contact” option; for one-third, the reason given was a desire to be seen in clinic, with a few giving the reasons of lack of consent (*n* = 2) and not wanting their child “labeled” or on a “register” (*n* = 2). The survey also investigated primary health-care use: 97% of respondents took their child to a general practitioner, and in 81% of cases, the general practitioner was aware of HIV in the family. Respondents were asked about contact from health services in the scenario of a potential health risk associated with exposure to a particular antiretroviral drug being identified: 84% indicated that they should be informed of such a risk even in the absence of a treatment or intervention, 14% that they should only be informed where treatment or intervention was available, and the remainder did not want to be contacted in any circumstance.

**Table 1:** **Four follow-up options for HEU children explored in surveys of parents/carers and health-care professionals**.

Option	Description
Clinic contact	Annual follow-up visit at pediatric clinic with data (no names) sent to the NSHPC. Parents/carers would need to inform the clinic of contact detail changes
Telephone contact	Pediatric clinic staff would call parent/carer annually to collect brief information about the child’s general health. Responses entered on a form and sent to NSHPC (no names). Parents/carers would need to inform the clinic of contact detail changes
Postal contact	NSHPC would be provided with contact details of parent/carer by the clinic when the child was discharged from routine care. A short form on the child’s health would be sent to parent/carer annually for completion and return to NSHPC (no reference to HIV anywhere on the form or correspondence). Parents/carers would need to inform the NSHPC of contact detail changes
No direct contact	NSHPC uses child’s NHS number to link to routinely available health information. No further contact with clinic or NSHPC needed after child is discharged

The health-care professional survey was smaller, involving 40 respondents from hospitals across the UK (87% response rate), of whom half were pediatricians and 40% pediatric nurses. All considered at least one of the options acceptable; acceptability of clinic contact, telephone contact, postal contact, and no direct contact was 70, 58, 55, and 53%, respectively. Overall, 28% strongly objected to clinic and/or telephone contact (where the burden of contact lay with the pediatric clinic), with lack of clinic resources being the main reason for this objection.

### Use of National Routine Data to Monitor Death and Cancer in HEU Children in England and Wales

Synthesis of the results of both the CHART Study and the survey provides a complex and sometimes contradictory picture of potentially acceptable and feasible approaches for long-term monitoring of outcomes in HEU children. Although annual clinic-based follow-up was the most acceptable option to parents and carers in the survey, the CHART Study demonstrated that this approach failed as a robust and sustainable strategy for monitoring health of HEU children. Furthermore, such an approach is not practical, or indeed, justifiable for surveillance on a population level using NHS resources, particularly when considering the cumulative increase in the HEU population. It was therefore clear that another approach for long-term surveillance of the health of HEU children was needed, even if this only provided data on a limited range of outcomes. The majority of parents and carers in the survey had no strong objection to the “no direct contact” option, using record linkage to routine health data.

A pilot study to “flag” HIV-exposed infants also exposed to antiretroviral drugs in fetal life with national registers to obtain notifications of any death or cancer registrations had been carried out in the 1990s. However, this process relied on manual matching to identify the relevant birth registration records, due to limited availability of the unique NHS number within the NSHPC at the time, and progress was slow; fewer than 400 children born between 1996 and 1999 were flagged with this system. In 2002, the NHS “Numbers for Babies” initiative resulted in the new policy of NHS numbers being allocated at birth rather than at civil registration and, as a result, an increasing proportion of infants reported to the NSHPC from this time had NHS number reported. To capitalize on this, in 2005, a new protocol was established for flagging children born to mothers living with HIV in England and Wales. This was initially *via* the NHS Central Register, administered by the Office of National Statistics, but following national restructuring, is now conducted *via* the Health and Social Care Information Center (HSCIC) (16).

An encrypted dataset containing the NSHPC variables, including NSHPC study number and the variables above, is securely transferred to HSCIC. The flagging procedure uses a matching algorithm, necessary not only because the NSHPC does not collect names but also because it is not appropriate to rely solely on the NHS number because of the potential for transcription errors. Child date of birth, sex, and NHS number allow for the large majority of children to be matched to the central record, but other variables (maternal date of birth and partial postcode of residence at delivery) are required to match the remaining children. Infant birthweight may also be used in an additional component of the algorithm where matching is complicated by twins. The flag within the central record has a generic label with no mention of HIV. A pseudonymized listing, with NSHPC study number as the sole identifier, is subsequently returned to the NSHPC. Events (i.e., deaths or cancer) are notified to the NSHPC twice a year, and include date and cause of death, and year of diagnosis, site, and type of cancer for death and cancer registrations, respectively. In an evaluation of the flagging procedure conducted on nearly 3000 children born 2001–2004 in England and Wales, flagging was possible in 95% of cases (16).

Currently, there are about 8000 uninfected infants born in England and Wales flagged, 97% born between 2001 and 2009, and the remainder earlier. This represents about half of all HEU children born in England and Wales to date. The NSHPC is currently working on flagging those born more recently and on assessing the event notifications.

In terms of monitoring the long-term health of HEU children, a drawback of our flagging approach is that death and cancers are the only available events, and this does not offer the opportunity to explore other morbidities, particularly those that may be associated with mitochondrial toxicity. There are possible options for additional record linkage studies, for example, with hospital episode statistics, in order to investigate whether any clinical outcomes are associated with *in utero* exposure to ART and maternal HIV. Another approach might be to conduct record linkage with results from the national program on newborn screening for inborn errors of metabolism, as was recently carried out in New York State (17), with the aim of determining whether the pattern of metabolic disorders is suggestive of mitochondrial toxicity. The feasibility and acceptability of such approaches need to be evaluated in the UK setting.

The benefits of our approach include good contemporaneous ascertainment of exposure to antiretroviral drugs (although direct adherence data are not collected, maternal viral load can be used as a proxy marker of adherence) and availability of other maternal and perinatal variables from the NSHPC database. There is likely to be minimal case ascertainment bias, as death registration is mandatory, and English cancer registries have been shown to have ascertainment rates of around 98–99% (18).

The ethical and information governance issues around any flagging study are important, but particularly in the context of fetal exposure to maternal HIV and to antiretroviral drugs, where there are concerns regarding disclosure and confidentiality within a family as well as in general. The original flagging study had independent approval from the relevant national body (the Patient Information Advisory Group), but is now a recognized component of the NSHPC as a whole, and covered with NSHPC ethical approval and approvals for use of non-consented patient identifiable data. The flagging process has been assessed as acceptable against NHS Information Governance policies and standards. Another important issue relates to the communication with mothers, fathers, children, and young people regarding safety monitoring, in order to reassure rather than provoke anxiety. The survey results demonstrate that, unsurprisingly, there was support from parents with respect to the need for monitoring (15), although this survey was conducted more than a decade ago and attitudes may have changed in the interim.

As mentioned above, we are currently working on extending the flagging to include the most recent birth cohorts of HEU children and on updating the previous results (16). No signal has yet been identified that raises concern about fetal exposure to ART with respect to cancer or death. However, consideration needs to be given to the necessary steps should a signal become apparent, for example, if rates for a specific cancer or cancer site were recorded at a higher rate than in the same age group in the general population.

## Approaches to Monitoring Outcomes in HEU Children in Other Countries

The French Perinatal Cohort (ANRS-Enquête Périnatale Française, EPF) has also used the approach of record linkage with national cancer registers to explore outcomes in HEU children. The EPF is a consented cohort study, with HEU children routinely followed up until age 2 years, after which there is a spontaneous pharmacovigilance reporting system with investigators reporting any known severe events (19). As for the NSHPC, the EPF does not collect names, and used child date and place of birth and sex to identify potential matches on the cancer registries, with manual checking. In addition to the registry linkage and spontaneous reporting, a specific information campaign was conducted to elicit spontaneous notification by a network of pediatric cancer specialists. No evidence of an overall increased cancer risk was found for HEU children born between 1984 and mid-2007 (median age, 5 years), using national rates as a reference; 5 of the 10 cancer cases were central nervous system malignancies, with a standardized incidence ratio of 3.1 (95% confidence interval 1.0, 7.2; *p* = 0.05).

Unlike the UK, a cohort study approach to monitor the health of HEUs has been successfully undertaken in the United States. The Pediatric HIV/AIDS Cohort Study (PHACS) Surveillance Monitoring of Antiretroviral Toxicity (SMART) protocol was established in 2005 and follows two cohorts of HEU children, one closed to new enrollments that include over 1200 children and the other continuing to enroll HEU infants. The SMART study uses a trigger-based approach to explore possible signals, whereby more rigorous, pre-defined investigations in individual children are prompted if predetermined clinical or laboratory thresholds are met, with a recent analysis reassuringly showing no association between meeting a case criteria and any antiretroviral class, regimen, or individual drug (20).

## Conclusion

The problems with recruitment and retention that were encountered within the CHART Study demonstrate that comprehensive, clinic-based follow-up is not a feasible approach for long-term assessment of the increasing number of HEU children in the UK. Although this Study was conducted more than 10 years ago, it likely that many of these problems would remain an issue today, particularly limited capacity at pediatric clinics and family mobility. It will therefore be important to continue flagging HEU children in cancer and death registries as one approach to monitoring for specific adverse outcomes of exposure to ART, particularly late outcomes that may not become apparent until late childhood, adolescence, or adulthood. The experience regarding *in utero* exposure to diethylstilbestrol (DES) underscores the need for long-term surveillance into adulthood, with the substantially and significantly elevated risk of rare cervical/vaginal cancers in daughters of women taking DES in pregnancy not identified until two or more decades after exposure (21–23). Future research strategies to address other health outcomes, including adverse events potentially related to mitochondrial/metabolic toxicity, in HEU children are being discussed, to capitalize on the NSHPC as a comprehensive, national surveillance anonymized record of HEU infants born to diagnosed women and the current and potential availability of electronic health records in the UK for record linkage.

## Author Contributions

CT and PT both contributed to the conception of the work. CT drafted the manuscript with PT revising critically for important intellectual content. CT and PT both have given approval for the final version and will both be accountable for all aspects of this work.

## Conflict of Interest Statement

No payments or support were received for the writing of this article. Other financial relationships between the authors and other entities are as follows: Public Health England (PT and CT), Medical Research Council (CT), Abbvie (PT and CT), EU FP7 (CT), PENTA Foundation (PT and CT), and National Institute for Health Research Biomedical Research Centres funding scheme.
